# Improved Characterisation of Vegetation and Land Surface Seasonal Dynamics in Central Japan with Himawari-8 Hypertemporal Data

**DOI:** 10.1038/s41598-019-52076-x

**Published:** 2019-10-30

**Authors:** Tomoaki Miura, Shin Nagai, Mika Takeuchi, Kazuhito Ichii, Hiroki Yoshioka

**Affiliations:** 1 Department of Natural Resources and Environmental Management, University of Hawai’i at Mānoa, 1910 East-West Road, Honolulu, Hawaii 96822 USA; 2 Research Institute for Global Change, Japan Agency for Marine-Earth Science and Technology, 3173-25 Showa-machi, Kanazawa-ku, Yokohama, 236-0001 Japan; 3 Center for Environmental Remote Sensing, Chiba University, 1-33 Yayoi-cho, Inage-ku, Chiba, 263-8522 Japan; 4 Department of Information Science and Technology, Aichi Prefectural University, 1522-3 Ibaragabasama, Nagakute, 480-1198 Japan

**Keywords:** Ecosystem ecology, Phenology

## Abstract

Spectral vegetation index time series data, such as the normalized difference vegetation index (NDVI), from moderate resolution, polar-orbiting satellite sensors have widely been used for analysis of vegetation seasonal dynamics from regional to global scales. The utility of these datasets is often limited as frequent/persistent cloud occurrences reduce their effective temporal resolution. In this study, we evaluated improvements in capturing vegetation seasonal changes with 10-min resolution NDVI data derived from Advanced Himawari Imager (AHI), one of new-generation geostationary satellite sensors. Our analysis was focused on continuous monitoring sites, representing three major ecosystems in Central Japan, where *in situ* time-lapse digital images documenting sky and surface vegetation conditions were available. The very large number of observations available with AHI resulted in improved NDVI temporal signatures that were remarkably similar to those acquired with *in situ* spectrometers and captured seasonal changes in vegetation and snow cover conditions in finer detail with more certainty than those obtained from Visible Infrared Imaging Radiometer Suite (VIIRS), one of the latest polar-orbiting satellite sensors. With the ability to capture *in situ*-quality NDVI temporal signatures, AHI “hypertemporal” data have the potential to improve spring and autumn phenology characterisation as well as the classification of vegetation formations.

## Introduction

Spectral vegetation index (VI) time series data have widely been used to monitor and characterise the Earth’s vegetative cover and its dynamics from regional to global scales^1–3^. They have been instrumental in the recent advancement of our understanding of vegetation-climate interaction^4–7^. VI time series datasets utilized in most of these studies were those derived from moderate resolution, polar-orbiting satellite sensors, such as Advanced Very High Resolution Radiometer (AVHRR), Moderate Resolution Imaging Spectroradiometer (MODIS), and VEGETATION.

The utility of these VI time series datasets in characterizing vegetation dynamics is often constrained by clouds. While a moderate resolution, polar-orbiting satellite sensor typically acquires one or two day-time observations per day, this observation frequency is only enough to generate “clear-sky” VI time series data at a weekly, 10-day, bi-weekly, or 16-day temporal resolution due to the frequent cloud cover^8,9^. The actual temporal resolution of those VI datasets can even be lower during the vegetation growing season^10,11^, which could then lower the accuracy of satellite-derived vegetation seasonal information^12^. Nagai *et al*.^10^, for example, reported that there could only be two or three clear-sky observations available during a two-week leaf expansion period in Japan even when data from the Terra and Aqua MODIS sensors were combined.

A new generation of geostationary satellite sensors have been launched during the last decade and planned for launch (see Supplementary Table S1). These sensors are capable of imaging an Earth’s hemisphere at 10–15 min intervals and equipped with the spectral bands suitable for the derivation of VIs, thus, potentially serving as another significant data source for the studies of vegetation dynamics^13^. The first of these was Spinning Enhanced Visible and Infrared Imager (SEVIRI) on Meteosat Second Generation (MSG) of which 3 km spatial resolution data became available in 2004^14^. All the others were either launched during the last five years or planned for launch in the next few years, and provide higher spatial resolution data (0.5–1 km) than SEVIRI, including Advanced Baseline Imager (ABI) on Geostationary Environmental Satellites (GOES)−16 and −17^15,16^, Advanced Geosynchronous Radiation Imager (AGRI) on Fengyun (FY)−4A^17,18^, Advanced Meteorological Imager (AMI) on Geostationary Korea Multi-Purpose Satellite (Geo-KOMPSAT)−2A, Advanced Himawari Imager (AHI) on Himawari-8 and-9^19^, and Flexible Combined Imager (FCI) on the Meteosat Third Generation Imaging (MTG-I) satellites^20^.

A series of studies by Fensholt *et al*.^13,21,22^ investigated the degree of improvement in the temporal resolution of the Normalized Difference Vegetation Index (NDVI) over Africa soon after SEVIRI data became available. Fensholt *et al*.^13^ showed a 79% increase in the number of cloud free days with SEVIRI (82 days) in comparison to MODIS (47 days) for the 2004 growing season in Senegal. They suggested that the period of compositing to produce continental-scale, cloud-free NDVI products could be reduced to 5 days with SEVIRI 15-min data from 10:00 to 14:00 over Africa. Another study by Fensholt *et al*.^21^ suggested 3 days as the shortest compositing period to produce cloud-free NDVI products over a cloud-prone region of West Africa when SEVIRI data from 10:00 to 16:00 were used. Fensholt *et al*.^22^ also examined the potential to standardize SEVIRI NDVI time series data to a fixed sun-view geometric condition with the bidirectional reflectance distribution function (BRDF) method published later by Proud *et al*.^23^ and found 5 days as the minimum to obtain robust results and to yet monitor short-term land surface changes.

Several studies have reported the utility of higher temporal resolution VI time series data from geostationary satellite sensors in characterizing vegetation seasonal changes^24^. Sobrino *et al*.^25^ was probably the first study that applied the NDVI dataset from a geostationary satellite sensor for the analysis of vegetation phenology. They found that the SEVIRI-retrieved green-up and brown-down dates were more accurate than the MODIS counterparts using a pan-European ground phenology record as a reference. High temporal resolution NDVI time series data derived from Geostationary Ocean Color Imager (GOCI) onboard the Communication Ocean and Meteorological Satellite (COMS)^26^ were effectively utilized to predict paddy rice yields in North Korea, a data-sparse and difficult-to-approach region^27^. Very recently, Yan *et al*.^28^ reported the first application of Himawari-8 AHI two-band Enhanced Vegetation Index (EVI2) time series data to land surface phenology in Northern and Central Japan. They found that AHI EVI2 higher temporal resolution data only helped improve the characterization of spring phenology in comparison to the MODIS counterpart. Yan *et al*.^28^ simply used 3-day temporal compositing to derive the AHI EVI time series data in their study, most likely following^21^ and^24^, but the resultant EVI2 time series data still contained a significant number of suspicious observations (see Fig. 8 in^28^).

In this study, we evaluated improvements in characterising vegetation and land surface dynamics with the AHI 10-min resolution NDVI data in Central Japan. The Central Japan area is subject to a series of unique seasonal events, including the monsoon and typhoon seasons, that bring about frequent and/or persistent cloud cover. It is of interest to understand how much the AHI geostationary satellite data could increase the temporal resolution of NDVI time series data under the influences of those geographically unique weather events. Our analysis was focused on continuous monitoring sites, representing three major ecosystems in the region (see Supplementary Fig. S1), where *in situ* time-lapse digital images documenting sky and surface vegetation conditions were available through the Phenological Eyes Network (PEN)^29^ for the verification purpose. AHI NDVI results were compared with those obtained from one of the latest polar-orbiting satellite sensors, Visible Infrared Imaging Radiometer Suite (VIIRS) onboard the Suomi-National Polar-orbiting Partnership (S-NPP).

## Results

### AHI NDVI temporal signatures

**Figure 1:**
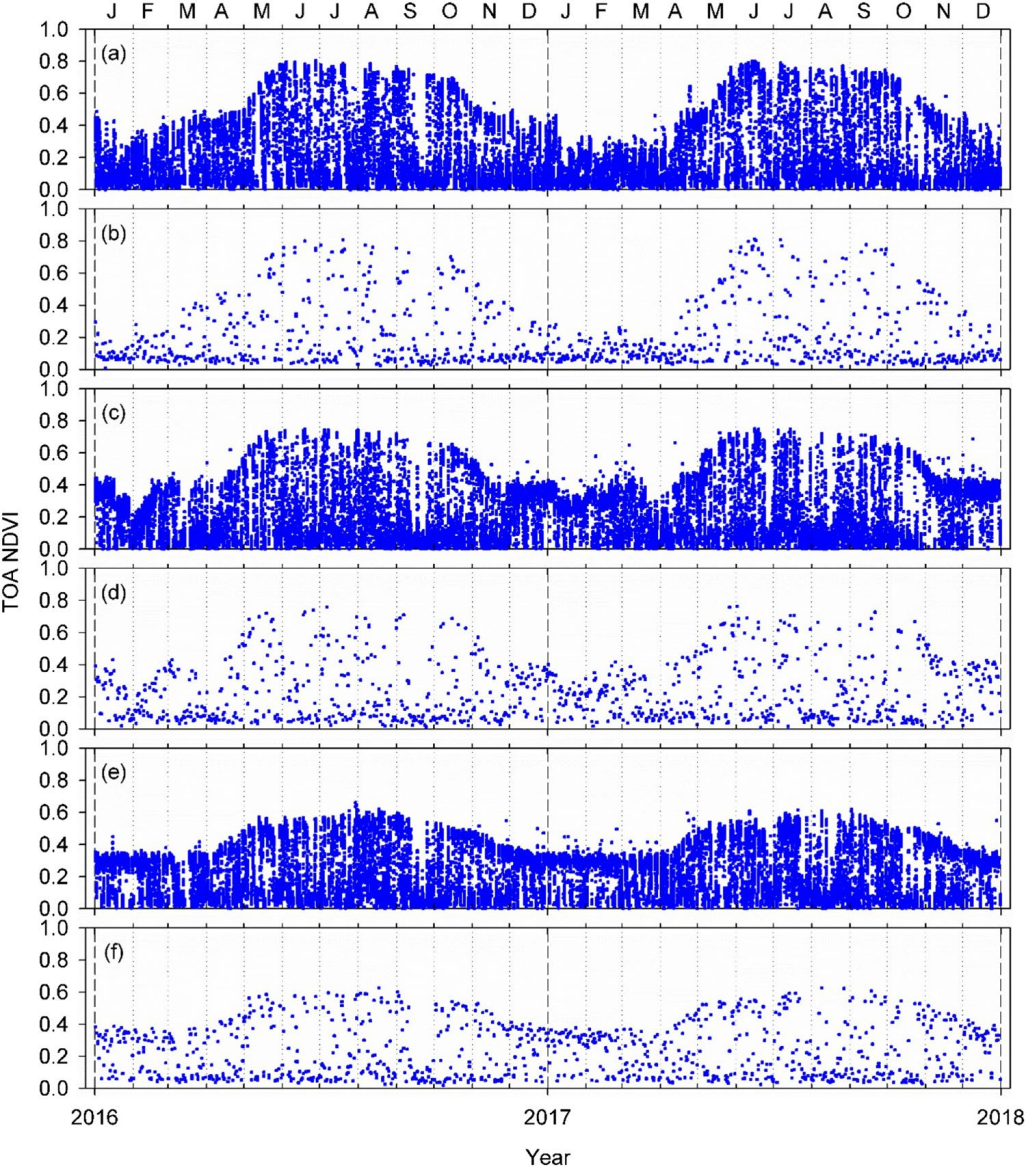
NDVI temporal profiles for the study sites: (**a**) AHI and (**b**) VIIRS for Takayama (TKY), (**c**) AHI and (**d**) VIIRS for Fujihokuroku (FHK), and (**e**) AHI and (**f**) VIIRS for Terrestrial Environment Research Center of University of Tsukuba (TGF).

We began our analysis by comparing AHI NDVI temporal profiles to those of VIIRS using the PEN-derived *in situ* phenology information as a reference (see Supplementary Table S2). The extracted AHI and VIIRS NDVI temporal profiles for the three study sites are shown in Fig. 1. All of the observations, including cloud-contaminated ones, were included in these plots. Since cloud contamination lowers the NDVI, cloud-contaminated NDVI data can be seen as those scattering below the upper envelopes of the NDVI temporal profiles which in turn most likely consisted of cloud-free pixels^30,31^. A small number of AHI NDVI observations that scattered above the upper envelopes and that were seen more frequently in the winter period (e.g., Fig. 1c,e) were those located near cloud edges, where the brightness typically varies significantly over a short distance. Due most likely to very small band-to-band mis-registration of AHI red and NIR bands, AHI reflectance spectra of those pixels were distorted, resulting in unusual NDVI values.

**Figure 2:**
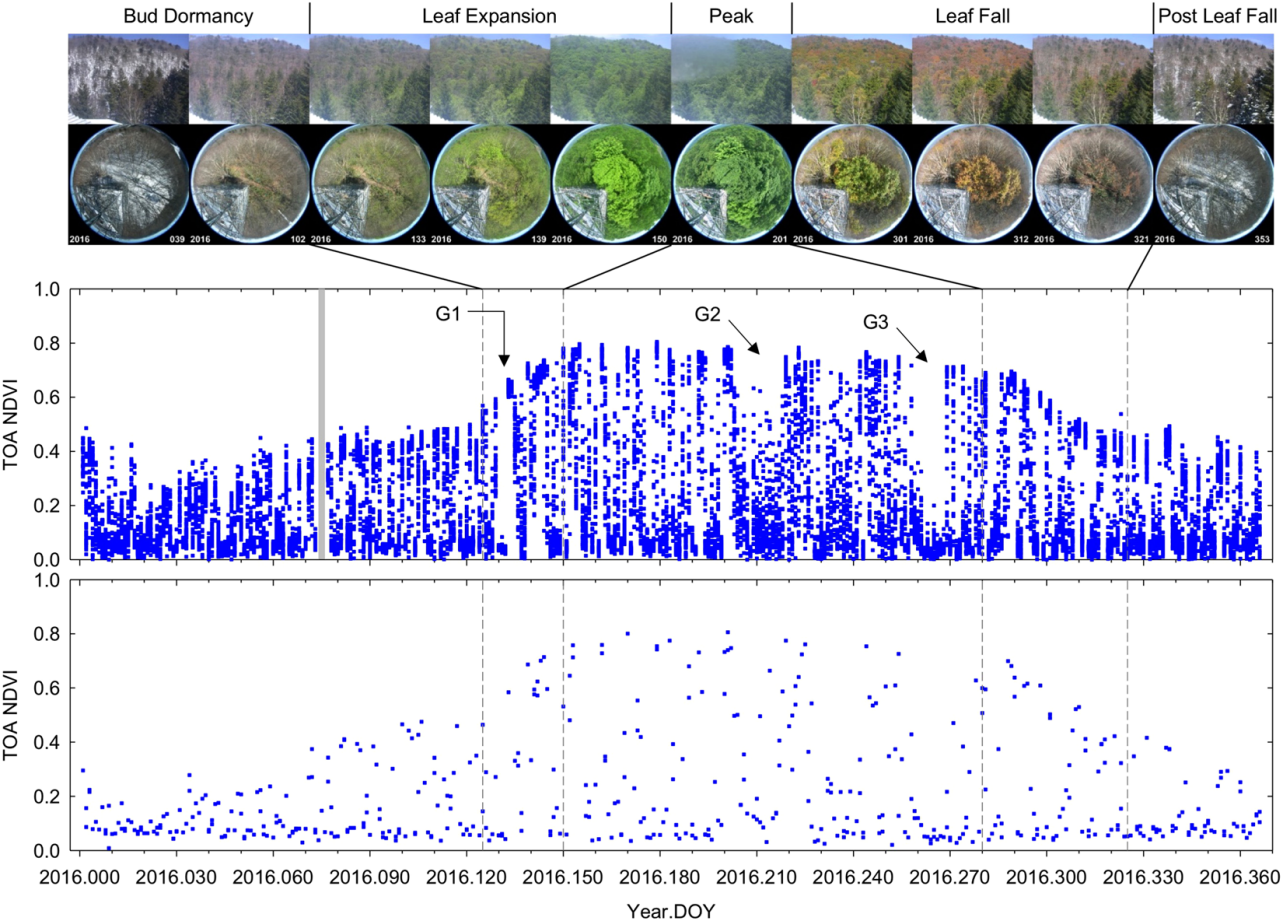
AHI NDVI temporal profile (middle) compared to the VIIRS counterpart (bottom) for the Takayama (TKY) site. Sample PEN *in situ* images representing five phenological stages are shown at the top. The numbers on the PEN images are their acquisition year (lower-left) and DOY (lower-right). The vertical dashed lines are phenological transition dates identified with PEN time-lapse images (see Supplementary Table S2), whereas the gray bar on the AHI NDVI plot corresponds to a two-day data gap.

Each VIIRS NDVI temporal profile was composed of ~500 observations per year (Fig. 1b,d,f). With its 10-min temporal resolution, in contrast, each of the AHI NDVI temporal profiles consisted of ~13,000 observations per year, 26 times more observations than VIIRS (Fig. 1a,c,e). This larger number of observations apparently made the AHI temporal profiles capture NDVI seasonal changes more clearly, or more certainly, than the VIIRS counterparts. In the VIIRS NDVI temporal profile of the Takayama deciduous broadleaf forest (TKY) site, for example, there was no observation with a high NDVI value for about a month from the middle of September to early October 2016 (Fig. 1b). The AHI NDVI temporal profile of the same site had approximately a 10-day period of continuously very low NDVI values earlier in this one-month period, but then had index values as high as those before the 10-day period for the rest of the period (Fig. 1a). Thus, with the AHI profile, one would be able to consider that the NDVI linearly decreased in this period with no abrupt vegetation change. Similar “gaps” were seen for the other two sites and they were associated with the passage of Typhoon-16 (Malakas) as described later in this section (Fig. 2). For the Fujihokuroku deciduous needleleaf forest (FHK) site, as another example, the VIIRS NDVI varied largely during the 2016 and 2017 summer (June-September) seasons, making it difficult to depict an cloud-free NDVI temporal signature (Fig. 1d). The AHI NDVI also varied largely, but due to the availability of a larger number of observations one could depict its flat signature with a little uncertainty (Fig. 1c). Compared to the above two sites, the VIIRS and AHI NDVI temporal profiles were very similar both in general and in detail for the Terrestrial Environment Research Center (TGF) site (Fig. 1e,f, respectively), a grass field surrounded by complex mosaics of urban, crop and rice paddy fields, grassland, and deciduous broadleaf forest patches.

To examine AHI NDVI temporal profiles in more detail, the TKY AHI NDVI temporal profile of the year 2016 is plotted along with the VIIRS counterpart in Fig. 2. The AHI data had a much larger number of seemingly “cloud-free” observations available throughout the entire year and around each of the four phenological transition dates than the VIIRS data (Fig. 2).

The AHI temporal profile contained several distinctive gaps (or temporal signatures) associated with weather events due to its 10-min temporal resolution, which were not clearly discernable in the VIIRS temporal profile. First, a small gap is seen in the AHI profile from the Day of Year (DOY) 130 to DOY 134 (G1 in Fig. 2). This was attributed to 3 consecutive days of very low NDVI values, which were found due to thick cloud cover by visual inspection of AHI false color composite images for the period, followed by 2 consecutive days of very high NDVI values. Second, there was a ~20-day period without high NDVI values around DOY 210 (G2 in Fig. 2). Visual inspection of AHI false color composite images over the period indicated that a large number of patchy clouds persisted over the study site during this period. Finally, there was another period only with low NDVI values (from DOY 259 to DOY 268) (G3 in Fig. 2), which was associated with the passage of Typhoon-16 (Malakas) (see Supplementary Fig. S2 for 2017).

**Figure 3:**
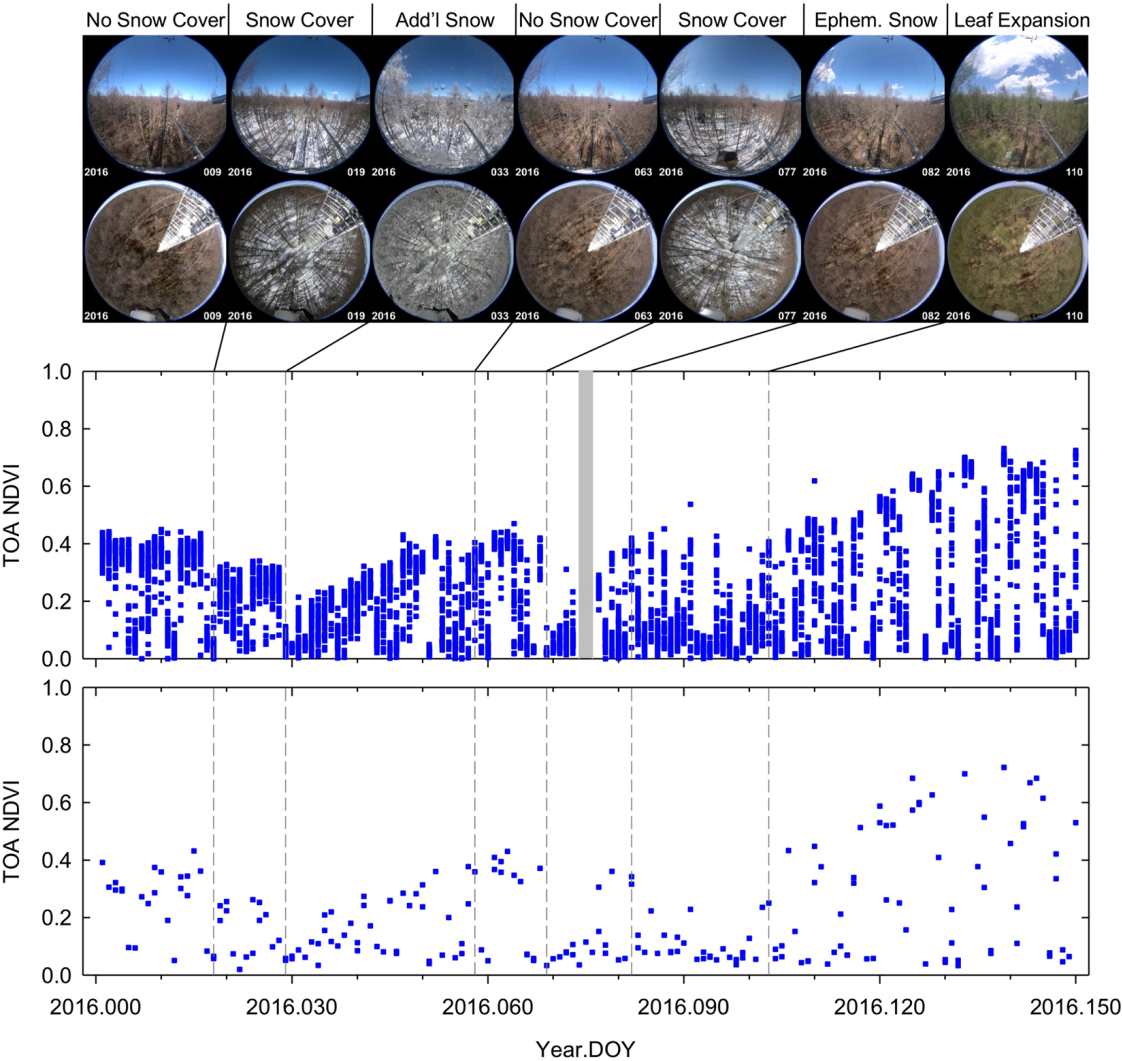
AHI NDVI seasonal changes (middle) during the first five months of the year 2016 for the Fujihokuroku (FHK) site. Plotted at the bottom is the VIIRS counterpart for comparison. Representative PEN *in situ* images for every distinctive snow cover condition are shown at the top. The numbers on the PEN images are their acquisition year (lower-left) and DOY (lower-right).

The AHI NDVI temporal profiles also depicted the changes in snow cover much better than the VIIRS counterparts. In Fig. 3, the AHI NDVI temporal profile for the FHK site is plotted along with the VIIRS counterpart and PEN images for the first 5 months of the year 2016. Based on the PEN image inspection, there was no snow cover for the first 17 days of the year 2016, snow covered the forest floor on DOY 18 and remained for the following 10 days until another snow fall accumulated more snow not only on the ground, but also on tree branches on DOY 29. The snow cover gradually melt over the next 30 days until they completely disappeared on DOY 58. It snowed again on DOY 69, but it melted during the following days until it completely disappeared again on DOY 82. Trees remained dormant though a couple of ephemeral snow cover occurred for a day or two until the start of leaf expansion on DOY 103. The AHI NDVI temporal dynamics corresponded exactly to these snow cover changes. The VIIRS NDVI was continuously very low from DOY 82 to DOY 105 when the site was subject only to a couple of ephemeral snow cover, making it difficult to detect/extract the date of the start of leaf expansion from the VIIRS temporal profile (see Supplementary Fig. S3 for 2017).

### Frequency analysis

**Figure 4:**
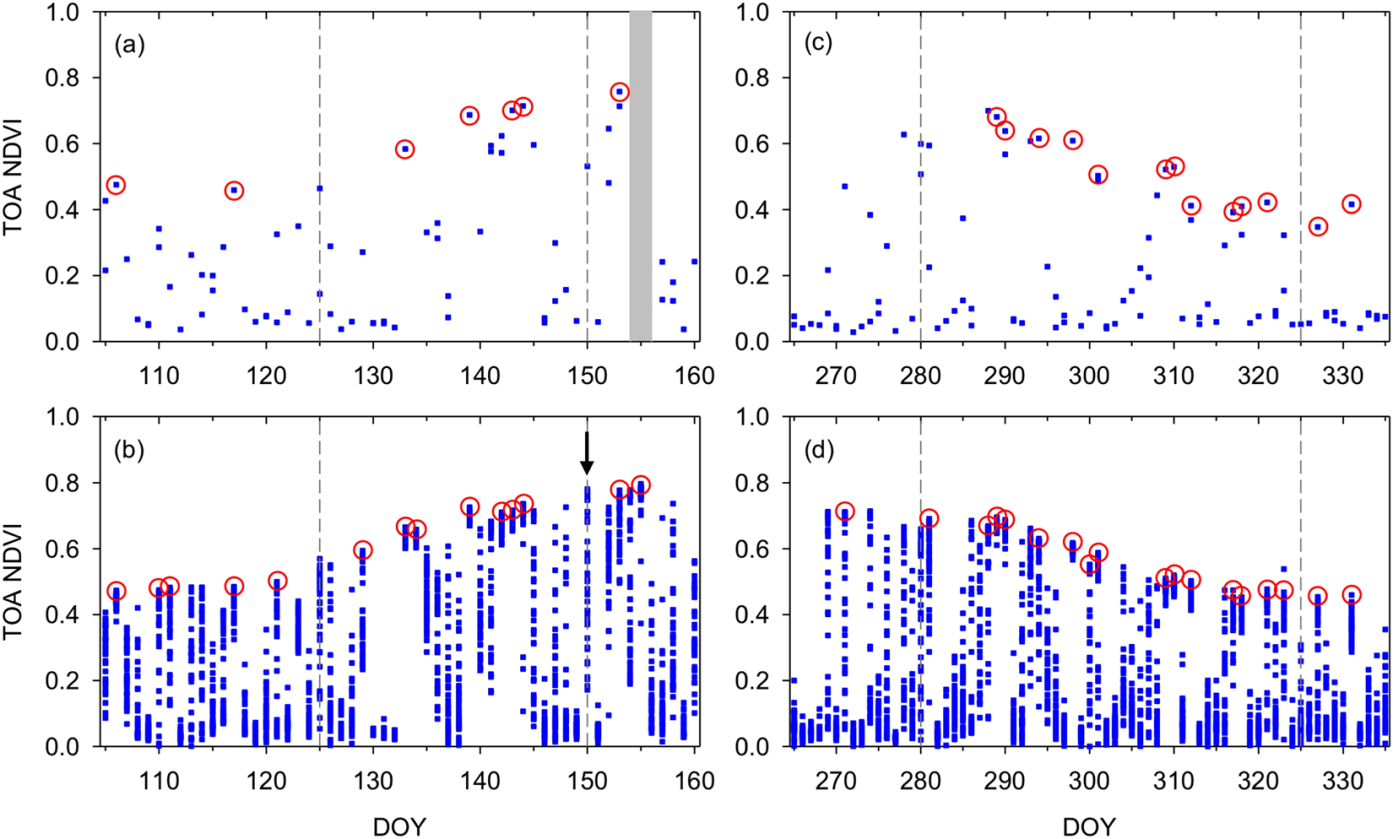
VIIRS and AHI NDVI data over spring green-up and fall brown-down periods for the Takayama (TKY) site: (**a**) VIIRS and (**b**) AHI for the green-up season, and (**c**) VIIRS and (**d**) AHI for the brown-down period.

We then examined an improvement in the available number of cloud-free observations with AHI high-temporal resolution data using PEN sky images as a reference. For each AHI observation, the PEN sky image acquired at the same or nearest time to the observation was selected as the reference. In Fig. 4, VIIRS and AHI NDVI data during the 2016 green-up and brown-down periods are compared for the TKY site. Here, the green-up and brown-down periods were defined as the period from the start to end of leaf expansion and that from the start to end of leaf fall, respectively (see Supplementary Table S2). Those observations confirmed as “cloud-free” by PEN *in situ* sky images are indicated by the red circles in the figure. The red circles are placed only on the maximum NDVI values when multiple “cloud-free” observations were found on the same day.

For the green-up period, whereas VIIRS had 4 cloud-free observation days, AHI had 7 days with at least one cloud-free observation (Fig. 4a,b, respectively). For the brown-down period, VIIRS had 11 days with at least one cloud-free observation, but AHI had 15 days with at least one cloud-free observation (Fig. 4c,d, respectively). Furthermore, VIIRS had only two cloud-free observation from DOY 105 to DOY 132, but AHI had at least one cloud-free observation on 5 days during the same period (Fig. 4a,b, respectively). Similarly, VIIRS had no cloud-free observations from DOY 265 to DOY 288, whereas AHI had 3 days with cloud-free observations (Fig. 4c,d, respectively) (see Supplementary Figs S4–S8 for the 2017 TKY and other two sites).

**Figure 5:**
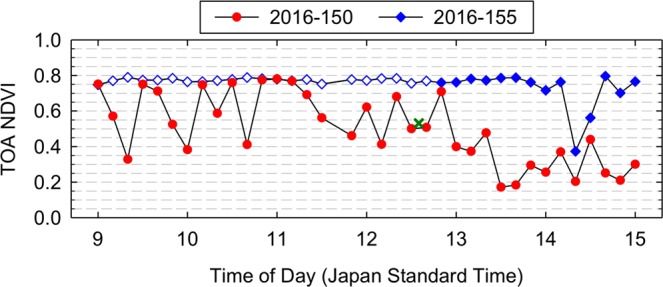
AHI NDVI diurnal time series plots of two different days for the Takayama (TKY) site. The blue open diamonds for DOY 155, 2016 represent “cloud-free” observations confirmed by PEN sky images. The “X” mark represents the VIIRS NDVI value for DOY 150, 2016.

**Figure 6:**
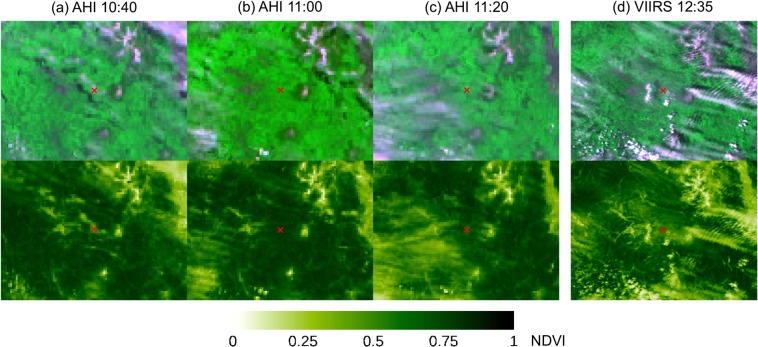
AHI and VIIRS subset false color composite (top) and NDVI (bottom) images over the Takayama (TKY) site for DOY 150, 2016. The false color composite images were made by assigning the red, NIR, and green bands to the red, green, and blue color planes. The color scale bar placed below the images is for the NDVI.

There existed several days that did not include any confirmed cloud-free observations, but whose maximum NDVI values were similar to those of nearby cloud-free observations, particularly in AHI NDVI, e.g., DOY 113, 114, 126, 141, 145, and 150 for the green-up period (see Fig. 4b). In Fig. 5, AHI NDVI data of DOY 150 are plotted as a 10-min time series along with those of DOY 155. The NDVI of DOY 155 changed little from 9:00 to 12:40 all of which observations were confirmed “cloud-free” by the PEN sky images, and the NDVI of DOY 150 was as high as that of DOY 155 for five different time periods during the morning (i.e., 9:00, 9:30, 10:10, 10:40, and 10:50–11:10) (Fig. 5). AHI subset time series images over the TKY site around 11:00 on DOY 150, 2016 showed the passages of several clouds and that there was no cloud cover over the TKY site exactly at 11:00 (Fig. 6a–c). VIIRS data were acquired at 12:35 on the day and the subset image over the TKY site showed clouds over the site (Fig. 6d) (See Supplementary Fig. S9 for DOY 113, 114, 126, 141, and 145 in 2016, and three additional days in 2017).

The mean numbers of days with cloud-free observations in AHI and VIIRS data confirmed with PEN sky images are summarized for the three study sites in Table 1 (see Supplementary Table S3 for the individual years). The green-up and brown-down periods for the TGF site were determined to enclose the periods of the increasing and decreasing NDVI, respectively, as observed in the AHI and VIIRS temporal profiles extracted over the site (i.e., Fig. 1e,f). For all the five phenological periods for all the three sites, AHI had larger numbers of days with cloud-free observations than VIIRS (Table 1). Whereas VIIRS had 4–5.5 days with cloud-free observations, AHI had 5.5–10 days with cloud-free observations, 1.6–1.8 times higher numbers for the green-up period. For the brown-down period, AHI had 1.4–1.6 times higher numbers of cloud-free observation days than VIIRS. Differences in the number of cloud-free observation days between AHI and VIIRS were the highest for the peak period, followed by the pre-green-up period, and the lowest in the post-brown-down period. Approximately, a day without cloud-contaminated observations can be expected every 4–6 days with AHI, but every 7–10 days with VIIRS for the green-up season, and a day without cloud-contaminated observations can be expected every 4 and 6 days with AHI and VIIRS, respectively, for the brown-down season (Table 1). A day with cloud-free observations can be expected at much higher frequencies with AHI (every 9–35 days) than with VIIRS (every 19–106 days) for the peak period, but at nearly the same frequencies with AHI and VIIRS during the post-brown-down period (every 2–6 days).

**Table 1:** Number of Days with Cloud-free Observations.

Period	Site	Range (DOY)	Number of Days with Cloud-free Obs.		Percentage (%)		Frequency		Ratio
			AHI	VIIRS	AHI	VIIRS	AHI	VIIRS	
Pre-green-up	TKY	1–127	31	17	24	13	4.2	7.6	1.8
	FHK	1–106	40	21.5	39	21	2.6	4.9	1.9
	TGF	1–90	50	34.5	56	39	1.8	2.6	1.4
Green-up	TKY	128–152	6.5	4	27	16	3.8	6.1	1.6
	FHK	107–138	5.5	3.5	17	11	6.0	9.5	1.6
	TGF	91–135	10	5.5	23	13	4.4	7.9	1.8
Peak	TKY	153–275	14.5	7.5	12	6.2	8.5	19	1.9
	FHK	139–281	4.5	1.5	3.2	1.1	35	106	3.0
	TGF	136–240	10.5	2.5	10	2.4	11	43	4.2
Brown-down	TKY	276–322	15	10	32	22	3.1	4.7	1.5
	FHK	282–332	17.5	11	34	22	3.1	4.6	1.6
	TGF	241–330	24	18	29	21	3.5	4.7	1.4
Post-brown-down	TKY	323–365	8.5	7.5	20	17	5.1	5.8	1.1
	FHK	333–365	22	15	68	48	1.5	2.1	1.4
	TGF	331–365	24.5	18.5	70	53	1.4	1.9	1.3

The percent values were calculated by dividing the number of days with cloud-free observations by the total number of days (with PEN sky images available), whereas the frequency values were obtained by dividing the latter by the former. The last column contains the ratio of the AHI percent value to that of VIIRS. All of values in the table are the average of the year 2016 and 2017 values (see Supplementary Table S3).

**Figure 7:**
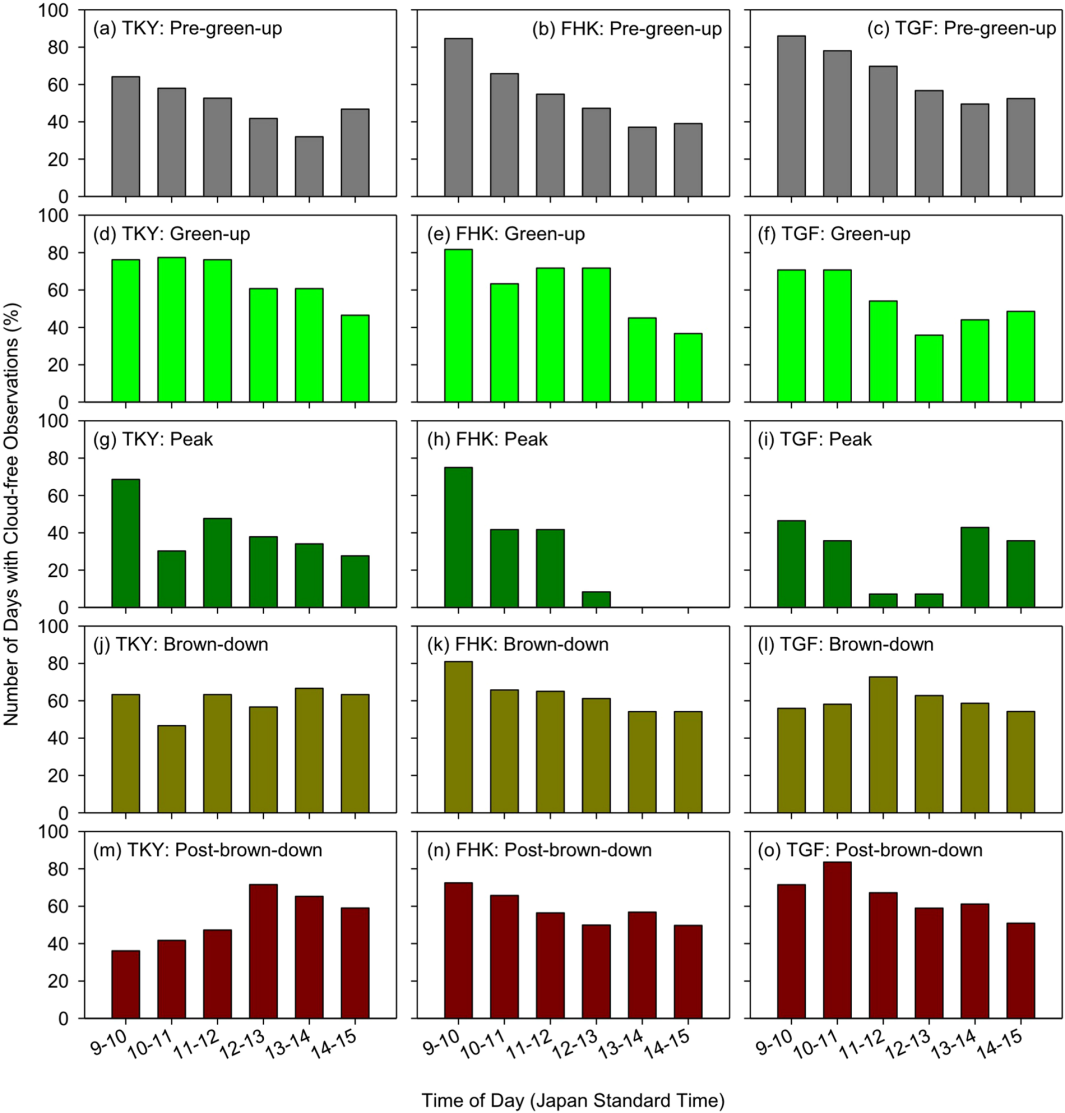
Diurnal distribution of AHI cloud-free observations. The percent values (the means of 2016 and 2017) were obtained for every one-hour period by dividing the number of cloud-free days for the specific one-hour period by the number of days with cloud-free observations anytime between 9:00 and 15:00.

Lastly, we examined the relationship of the number of AHI cloud-free observations with the time of day for the five phenological periods (Fig. 7). Those days when, at least, one PEN-confirmed, cloud-free AHI observation was found were used in this analysis. Not all AHI cloud-free observations came from any single one-hour period, but across the 6-hour period, except for the peak season at FHK and TGF. For all the three sites, in general, the highest number of AHI cloud-free observations were available in early morning (9–10) for the pre-green-up, green-up, and peak periods (Fig. 7a–i). The same, but weaker trend was observed for the brown-down and post-brown-down periods for the FHK site (Fig. 7k,n). For the TKY and TGF sites, AHI cloud-free observations were more equally distributed across the 6-hour period for the brown-down period (Fig. 7j,l), and came more from the afternoon and from the morning, respectively, for the post-brown-down period (Fig. 7m,o).

## Discussion

In this study, we evaluated AHI 10-min resolution NDVI data with respect to the available number of cloud-free observations and associated quality improvement in the NDVI temporal signatures using continuous monitoring sites in Central Japan where *in situ* time-lapse digital images were available. The very large number of observations available with AHI resulted in improved NDVI temporal signatures, allowing to capture seasonal changes in vegetation and snow cover conditions in more detail with more certainty than VIIRS NDVI. As shown for the African continent in previous studies^13,21^, the longer and more frequent observations per day with the AHI geostationary sensor allowed to obtain cloud-free observations on those days where only cloud-contaminated observations could be acquired with polar-orbiting satellite sensors. Our results at the three sites in Central Japan have demonstrated that AHI geostationary sensor data were able to provide one cloud-free observation day every 4–6 days during the green-up period and every 4 days during the brown-down period. Our results have also indicated that the benefit of AHI high temporal resolution data to phenology studies can be greater for the spring than for the fall as the increase in the number of days with cloud-free observation(s) with AHI was higher for the former than for the latter, supporting the results by Yan *et al*.^28^. In addition, persisting low NDVI values due to persistent cloud cover could appear as a discernable temporal signature in the AHI NDVI temporal profiles which could be advantageous as the impacts of weather events such as the passages of typhoons on vegetation condition could be assessed using this single dataset.

The AHI NDVI temporal profiles of TKY and FHK were remarkably similar to those acquired with *in situ* spectrometers^32–34^. Although the AHI NDVI temporal profiles contained several notable data gaps during the summer due to frequent or persistent cloud occurrences associated with geographically unique seasonal weather events (e.g., the passages of typhoons), these “hypertemporal” signatures of AHI NDVI have the potential not only to improve spring phenology characterization^28^, but also to discriminate among different vegetation formations and/or across different canopy layers as forest and grass species exhibit different NDVI temporal signatures^32^. Recently, a number of studies reported analysis results on autumn phenology. Compared with spring phenology, which has been well studied, autumn phenology is still poorly understood^35^. Several recent studies have shown the effects of weather conditions during the growth period on the end of growing season^36^, the interaction of the start of growing season and weather conditions impacting the end of growing season^37,38^, and the long-term legacy effect where trees that experienced a warm winter-spring season had earlier leaf-flushing not only in the same year, but also in the following year^38^. With the ability to capture *in situ* level NDVI temporal signatures with the shown improvement in the spring phenology, AHI hypertemporal data may serve as a unique dataset to contribute to detailed, improved analysis of autumn phenology. Several studies utilized the normalized difference water index (NDWI) or snow index (NDSI) computed from AHI 1.6 μm band in combination with one of AHI visible bands to identify snow cover as part of their AHI cloud detection algorithms^39,40^. AHI NDVI temporal signatures in combination with those of NDWI or NDSI may provide an additional information on vegetation phenology.

This study has also demonstrated the critical importance of continuously-acquired *in situ* time series validation data, such as time-lapse digital images as used in this study, tower reflectance measurements, and carbon flux measurements. VI temporal profiles derived from geostationary satellite data would show finer temporal signatures than those captured with polar-orbiting satellite sensors. As shown in this study, such fine temporal signatures could only be interpreted correctly with the aid of *in situ* measured validation datasets. High spatial resolution data of improved temporal resolution (e.g., Sentinel) may serve as an effective means to scale up the information obtained from *in situ* validation data to the geostationary satellite footprint for their time series validation^41,42^.

Whereas the fixed position of the AHI sensor makes it possible to provide finer temporal resolution VI profiles, the AHI’s view and azimuthal angles are constant at every location with constantly changing solar zenith and azimuthal angles at every location. AHI data from the early morning or late afternoon are acquired at larger SZA than those from polar-orbiting satellites at mid-latitudes. Likewise, the SZA diurnal variation of AHI data could be larger than their seasonal counterpart with their relative azimuth angles (RAA) very different from those encountered by polar-orbiting satellites^43^. Examining the NDVI variations with respect to these SZA and RAA variations is important to improve our understanding of the AHI NDVI behaviors. Normalizing this geometric source of variations would likely be a required pre-processing step^23,28^, particularly when the integration of multiple geostationary satellite data, or geostationary and polar-orbiting satellite data are considered.

## Materials and Methods

### Study sites

Three study sites were selected in this study, all located in Central Japan (see Supplementary Fig. S1). They represented three major ecosystems in the region: a cool-temperate, deciduous broadleaf forest (Takayama, TKY), a warm-temperate, deciduous needleleaf forest (Fujihokuroku, FHK), and a grassland (Terrestrial Environment Research Center of the University of Tsukuba, TGF). All these sites are part of the Phenological Eyes Network (PEN), “a network of long-term ground observation sites aimed at validating terrestrial remote sensing with a particular focus on vegetation seasonal changes (phenology)^29^,” as well as part of the AsiaFlux network (http://www.asiaflux.net/). The TKY and FHK sites are also part of the Japan Long-Term Ecological Research Network (JaLTER, http://www.jalter.org/en/).

The TKY site (36°08′46″N, 137°25′23″E, 1420 m above sea level) was a secondary deciduous broadleaf forest with the dominant canopy species being *Betula ermanii* (birch) and *Quercus crispula* (oak)^44^. The height of the tree canopy ranged from 13 to 20 m. The forest floor was covered by an evergreen dwarf bamboo, *Sasa senanensis*^44^. The annual mean air temperature and precipitation were 6.4 °C and 2075 mm, respectively (1994–2008)^45^. The snow period extended from early December to late April with the maximum snow depth being usually 1.0–1.5 m^45^.

At the FHK site (35°26′37″N, 138°45′53″E, 1106 m above sea level), the dominant overstory species was Japanese larch (*Larix kaempferi*), planted around 1950, with the canopy height ranging from 20 to 26 m, whereas the forest understory was predominantly buckler fern (*Dryopteris crassirhizoma*)^46^. For a period from 2006 to 2013, the annual mean air temperature and precipitation were 8.6 °C and 1848 mm, respectively^47^. For the period, the maximum snow depth occurred from January to February and ranged between 22 and 54 cm^47^. At both the TKY and FHK sites, their major forest covers were spatially extensive, but mixed with small patches of evergreen needleleaf forests^28^.

The TGF site (36°6′49″N, 140°5′42″E, 29 m above sea level) was a circular grass field with a diameter of 160 m^48^ and was surrounded by complex mosaics of urban, crop and rice paddy fields, grassland, and deciduous broadleaf forests^28^. The field consisted of a mixture of C3 and C4 grasses with the dominant species being *Solidago altissima*, *Miscanthus sinensis*, and *Imperata cylindrica*. The annual mean air temperature and precipitation were 14.1 °C and 1207 mm, respectively, over a period of 1982–2001.

While these sites differ in climate and elevation as described above, all the three sites experience the monsoon rainy season of Japan (“baiu”) every year^49^. Japan Meteorological Agency declares the baiu season every year. All the sites experienced the baiu season from early-June to late-July in 2016. The 2017 baiu season lasted about a month, but started in early-June, mid-June, and late-June for the TGF, FHK, and TKY sites, respectively. Japan also experiences at least one typhoon landfall per year^50^. There were six and four landfalling typhoons in 2016 and 2017, respectively (https://www.jma.go.jp/en/typh/index.html).

### AHI data

Himawari-8 AHI full-disk radiance data from January 1, 2016 to December 31, 2017 were obtained from the Center for Environmental Remote Sensing (CERES) at Chiba University, Japan. These data were precisely geo-rectified and remapped onto a 0.005-degree (red band) and 0.01-degree (blue, green, and NIR bands) linear latitude-longitude grid (http://www.cr.chiba-u.jp/databases/GEO/H8_9/FD/index.html).

These AHI data were first subset over a rectangular region covering the whole country of Japan (50°N–20°N and 120°E–150°E). The red band images were spatially aggregated into 0.01-degree pixels by taking arithmetic means of 2-by-2 pixels. They were then converted from at-sensor radiance to top-of-atmosphere (TOA) reflectance

1 $${\rho }_{b,i}^{TOA}=\frac{\pi {L}_{b,i}{d}^{2}}{{E}_{b}cos{\theta }_{i}}$$

where

${\rho }_{b,i}^{TOA}$ TOA reflectance for band *b* at pixel *i*

*L*_*b,i*_ At-sensor radiance for band *b* at pixel *i* (W/m^2^/sr/μm)

*E*_*b*_ Exo-atmospheric solar irradiance for band *b* (W/m^2^/μm)

*d* Earth-Sun distance (AU)

*θ*_*i*_ Solar zenith angle at pixel *i*

In deriving TOA reflectance, the Earth-Sun distance was calculated on a daily basis, whereas the solar zenith angle was calculated on a per-scene, per-pixel basis using an algorithm described in^51^. AHI view zenith and azimuth angles were computed for every pixel, but for one time^52^ by assuming that the movement of the Himawari-8 satellite position did not significantly impact AHI view zenith and azimuthal angles. Finally, the NDVI was computed from the derived red and NIR reflectances

2 $$NDVI=\frac{{\rho }_{{\rm{NIR}}}^{TOA}-{\rho }_{{\rm{red}}}^{TOA}}{{\rho }_{{\rm{NIR}}}^{TOA}+{\rho }_{{\rm{red}}}^{TOA}}$$

We retained AHI NDVI data collected between 9:00 and 15:00 Japan standard time (±3 hours of local noon) for analysis. TOA NDVI were shown to change little during this time period^21^, whereas the illumination geometry constantly changed throughout the period. For our study sites, the solar zenith angle (SZA) of AHI observations ranged from 12° to 75° with 44° being the average annually. Diurnally, they varied from 12° to 41° on the summer solstice, from 35° to 59° on the equinoxes, and from 59° to 75° on the winter solstice. AHI observations with SZA greater than 70° were only found close to 9:00 or 15:00 in the winter period (November – January), which were excluded from analysis in previous studies^22,23^, but included in this study.

### VIIRS data

S-NPP VIIRS TOA reflectance and geo-angle granule products were acquired from the NOAA Comprehensive Large Array-data Stewardship System (CLASS, https://www.class.noaa.gov/) for the same two-year period over the same geographic region. These products were all at a “validated” maturity status as defined by the Joint Polar-orbiting Satellite System Algorithm Engineering Review Board (AERB)^53,54^. Red and NIR (I1 and I2, respectively) TOA reflectances, solar and view zenith, and relative azimuth angle science data sets contained in these products were mosaiced and remapped onto a 0.00375-degree linear latitude-longitude grid on a per-orbit basis as VIIRS ground swaths from two adjacent orbits overlapped significantly (~30%). The NDVI was computed from the red and NIR reflectances using Eq. (2). For each site, data were extracted and averaged over a 2-pixel-by-2-pixel window to compensate for the spatial resolution difference.

### PEN *In Situ* sky and phenological images

*In situ* hemispherical sky images for the three study sites were obtained from the PEN data archive for the same two-year period of January 1, 2016 to December 31, 2017^55^. At each of the sites, these PEN sky images were acquired with an upward-looking camera system, namely, the automatic digital fish-eye camera (ADFC)^29^, mounted on the flux tower above the canopy surface layer of the site and configured to shutter at fixed time intervals during the daytime. At the TKY site, sky images were taken at 2-minute intervals from January 1 2016 through DOY 305, 2016 and at 3-minute intervals from DOY 306, 2016. At the FHK and TGF sites, sky images were taken every 10 and 3 minutes, respectively. Every hemispherical sky image was visually inspected for any cloud contamination and labeled as “clear” when no cloud was seen across the hemi-spherical field-of-view, thus, being conservatively estimated.

*In situ* time-lapse images of the canopy surfaces and landscapes of the TKY and FHK sites were also obtained from the PEN archive for the same two-year period^55^. At the TKY site, the canopy surface images were acquired with a downward-looking ADFC mounted on the flux tower at a height of 18 m, whereas the landscape images were taken over the south side of the flux tower with another ADFC installed on the rooftop of a nearby small building. At the FHK site, the canopy surface and landscape images were taken with a downward-looking ADFC attached on the flux tower at 32 m in height and a side-looking (northward) ADFC at a height of 22 m on the same tower. All the four cameras took images every 90 minutes during the daytime. The canopy and landscape images taken at local noon were used to identify the timing of phenophase transitions and snow cover for the two sites. *In situ* time-lapse images of the land surface and landscape of the TGF site were also available, but were not used in this study. They were setup only to capture the 160 m-diameter circular grass field and did not represent well the larger land surface area captured by the AHI pixel observations.

## Supplementary information

Supplementary Information

Supplementary Dataset

## Data Availability

The AHI, VIIRS, and PEN datasets generated and analysed during the current study are available from the corresponding author on reasonable request.

## References

[1] Myneni RB, Keeling CD, Tucker CJ, Asrar G, Nemani RR (1997). Increased plant growth in the northern high latitudes from 1981 to 1991. Nature.

[2] Zhang X, Liu L, Yan D (2017). Comparisons of global land surface seasonality and phenology derived from AVHRR, MODIS, and VIIRS data. J. Geophys. Res.-Biogeo..

[3] Zhang Y (2019). Mapping annual forest cover by fusing PALSAR/PALSAR-2 and MODIS NDVI during 2007–2016. Remote Sens. Environ..

[4] Ponce Campos GE (2013). Ecosystem resilience despite large-scale altered hydroclimatic conditions. Nature.

[5] Chen B, Xu G, Coops NC, Ciais P, Myneni RB (2016). Satellite-observed changes in terrestrial vegetation growth trends across the Asia-Pacific region associated with land cover and climate from 1982 to 2011. International Journal of Digital Earth.

[6] Seddon AWR, Macias-Fauria M, Long PR, Benz D, Willis KJ (2016). Sensitivity of global terrestrial ecosystems to climate variability. Nature.

[7] Saleska SR (2016). Dry-season greening of Amazon forests. Nature.

[8] Huete A (2002). Overview of the radiometric and biophysical performance of the MODIS vegetation indices. Remote Sens. Environ..

[9] Toté C (2017). Evaluation of the SPOT/VEGETATION Collection 3 reprocessed dataset: Surface reflectances and NDVI. Remote Sens. Environ..

[10] Nagai S (2011). The necessity and availability of noise-free daily satellite-observed NDVI during rapid phenological changes in terrestrial ecosystems in East Asia. Forest Science and Technology.

[11] Armitage RP, Ramirez FA, Danson FM, Ogunbadewa EY (2012). Probability of cloud-free observation conditions across Great Britain estimated using MODIS cloud mask. Remote Sens. Lett..

[12] Zhang X, Friedl M, Schaaf C (2009). Sensitivity of vegetation phenology detection to the temporal resolution of satellite data. Int. J. Remote Sens..

[13] Fensholt R, Sandholt I, Stisen S, Tucker C (2006). Analysing NDVI for the African continent using the geostationary Meteosat Second Generation SEVIRI sensor. Remote Sens. Environ..

[14] Schmetz J (2002). An Introduction to Meteosat Second Generation (MSG). Bull. Am. Meteorol. Soc..

[15] Schmit TJ (2005). Introducing the next-generation Advanced Baseline Imager on GOES-R. Bull. Am. Meteorol. Soc..

[16] Schmit TJ, Lindstrom SS, Gerth JJ, Gunshor MM (2018). Applications of the 16 spectral bands on the Advanced Baseline Imager (ABI). J. Operational Meteor..

[17] Yang J, Zhang Z, Wei C, Lu F, Guo Q (2016). Introducing the new generation of Chinese geostationary weather satellites, Fengyun-4. Bull. Am. Meteorol. Soc..

[18] Zhang P (2019). General comparison of FY-4A/AGRI with other GEO/LEO instruments and its potential and challenges in non-meteorological applications. Frontiers in Earth Science.

[19] Bessho K (2016). An introduction to Himawari-8/9 - Japan’s new-generation geostationary meteorological satellites. J. Meteorol. Soc. Japan.

[20] EUMETSAT. Meteosat Third Generation, https://www.eumetsat.int/website/home/Satellites/FutureSatellites/MeteosatThirdGeneration/ (2019).

[21] Fensholt R (2007). Comparisons of compositing period length for vegetation index data from polar-orbiting and geostationary satellites for the cloud-prone region of West Africa. Photogramm. Eng. Remote Sens..

[22] Fensholt R (2011). Analysing the advantages of high temporal resolution geostationary MSG SEVIRI data compared to Polar Operational Environmental Satellite data for land surface monitoring in Africa. In. J. Appl. Earth Obs. Geoinf..

[23] Proud SR (2014). The normalization of surface anisotropy effects present in SEVIRI reflectances by using the MODIS BRDF method. IEEE Trans. Geosci. Remote Sens..

[24] Yan D, Zhang XY, Yu YY, Guo W (2017). Characterizing land cover impacts on the responses of land surface phenology to the rainy season in the Congo basin. Remote Sensing.

[25] Sobrino JS, Julien Y, Soria G (2013). Phenology estimation from Meteosat Second Generation data. IEEE J. Sel. Topics Appl. Earth Observ..

[26] Choi J-K (2012). GOCI, the world’s first geostationary ocean color observation satellite, for the monitoring of temporal variability in coastal water turbidity. J. Geophys. Res.-Oceans.

[27] Yeom J-m (2018). Monitoring paddy productivity in North Korea employing geostationary satellite images integrated with GRAMI-rice model. Sci. Rep..

[28] Yan D (2019). Evaluating land surface phenology from the Advanced Himawari Imager using observations from MODIS and the Phenological Eyes Network. In. J. Appl. Earth Obs. Geoinf..

[29] Nasahara KN, Nagai S (2015). Review: Development of an *in situ* observation network for terrestrial ecological remote sensing: the Phenological Eyes Network (PEN). Ecol. Res..

[30] van Leeuwen WJD, Huete AR, Laing TW (1999). MODIS vegetation index compositing approach: A prototype with AVHRR data. Remote Sens. Environ..

[31] Chen J (2004). A simple method for reconstructing a high-quality NDVI time-series data set based on the Savitzky-Golay filter. Remote Sens. Environ..

[32] Motohka, T., Nasahara, K. N., Oguma, H. & Tsuchida, S. Applicability of Green-Red Vegetation Index for Remote Sensing of Vegetation Phenology. *Remote Sensing* **2** (2010).

[33] Muraoka H (2013). Spectral vegetation indices as the indicator of canopy photosynthetic productivity in a deciduous broadleaf forest. J. Plant Ecol..

[34] Ide R, Hirose Y, Oguma H, Saigusa N (2016). Development of a masking device to exclude contaminated reflection during tower-based measurements of spectral reflectance from a vegetation canopy. Agric. For. Meteorol..

[35] Gallinat AS, Primack RB, Wagner DL (2015). Autumn, the neglected season in climate change research. Trends Ecol. Evolut..

[36] Wu C (2018). Contrasting responses of autumn-leaf senescence to daytime and night-time warming. Nature Clim. Change.

[37] Xie Y, Wang X, Wilson AM, Silander JA (2018). Predicting autumn phenology: How deciduous tree species respond to weather stressors. Agric. For. Meteorol..

[38] Fu YSH (2014). Variation in leaf flushing date influences autumnal senescence and next year’s flushing date in two temperate tree species. Proc. Natl. Acad. Sci..

[39] Imai T, Yoshida R (2016). Algorithm theoretical basis for Himawari-8 cloud mask product. Meteorological Satellite Center Technical Note.

[40] Shang H (2017). Development of a daytime cloud and haze detection algorithm for Himawari-8 satellite measurements over central and eastern China. J. Geophys. Res.-Atmos..

[41] Arroyo-Mora JP (2018). Evaluation of phenospectral dynamics with Sentinel-2A using a bottom-up approach in a northern ombrotrophic peatland. Remote Sens. Environ..

[42] Vrieling A (2018). Vegetation phenology from Sentinel-2 and field cameras for a Dutch barrier island. Remote Sens. Environ..

[43] Adachi Y, Kikuchi R, Obata K, Yoshioka H (2019). Relative azimuthal-angle matching (RAM): A screening method for GEO-LEO reflectance comparison in middle latitude forests. Remote Sensing.

[44] Ohtsuka T (2005). Biometric based estimates of net primary production (NPP) in a cool-temperate deciduous forest stand beneath a flux tower. Agric. For. Meteorol..

[45] Noh NJ, Kuribayashi M, Saitoh TM, Muraoka H (2017). Different responses of soil, heterotrophic and autotrophic respirations to a 4-year soil warming experiment in a cool-temperate deciduous broadleaved forest in central Japan. Agric. For. Meteorol..

[46] Takahashi Y (2015). Characteristics of temporal variations in ecosystem CO2 exchange in a temperate deciduous needle-leaf forest in the foothills of a high mountain. Journal of Agricultural Meteorology.

[47] Teramoto M, Liang N, Zeng J, Saigusa N, Takahashi Y (2017). Long-term chamber measurements reveal strong impacts of soil temperature on seasonal and inter-annual variation in understory CO2 fluxes in a Japanese larch (Larix kaempferi Sarg.) forest. Agric. For. Meteorol..

[48] Akitsu T (2011). Long-term observation of seasonal and yearly variation of grassland by an automatic digital camera. Bull. Terrestrial Environ. Res. Center Univ. Tsukuba.

[49] Takayabu YN, Hikosaka K (2009). Statistical analysis of oceanic rainfall characteristics in the Baiu season utilizing TRMM PR data. J. Meteorol. Soc. Japan.

[50] Fudeyasu H, Hirose S, Yoshioka H, Kumazawa R, Yamasaki S (2014). A global view of the landfall characteristics of tropical cyclones. Tropical Cyclone Research and Review.

[51] Reda I, Andreas A (2004). Solar position algorithm for solar radiation applications. Solar Energy.

[52] Manago N (2016). Atmospheric correction of satellite image using python, Lecture series 2: Atmospheric correction with a standard atmosphere model. Journal of the Remote Sensing Society of Japan.

[53] Cao C, DeLuccia FJ, Xiong X, Wolfe R, Weng F (2014). Early on-orbit performance of the Visible Infrared Imaging Radiometer Suite onboard the Suomi National Polar-Orbiting Partnership (S-NPP) satellite. IEEE Trans. Geosci. Remote Sens..

[54] Wolfe RE (2013). Suomi NPP VIIRS prelaunch and on-orbit geometric calibration and characterization. J. Geophys. Res.-Atmos..

[55] Nagai, S. *et al*. 8 million phenological and sky images from 29 ecosystems from the Arctic to the tropics: the Phenological Eyes Network. *Ecol. Res*. (2018).

