# Supplementary material for: Improved Characterisation of Vegetation and Land Surface Seasonal Dynamics in Central Japan with Himawari-8 Hypertemporal Data
Source: Sci Rep. 2019 Oct 30;9:15692. doi: 10.1038/s41598-019-52076-x (PMC6821777; doi:10.1038/s41598-019-52076-x)

Supplementary Information for:

Improved Characterisation of Vegetation and Land Surface Seasonal Dynamics in Central Japan with Himawari-8 Hypertemporal Data

Tomoaki Miura*, Shin Nagai, Mika Takeuchi, Kazuhito Ichii, and Hiroki Yoshioka

Supplementary Table S1. Comparison of Select Characteristics of New-generation Geostationary Satellite Sensors

<table>
<tr><td>Sensor</td><td colspan="2">SEVIRI</td><td colspan="2">ABI</td><td colspan="2">AGRI</td><td colspan="2">AMI</td><td colspan="2">AHI</td><td colspan="2">FCI</td></tr>
<tr><td>Platform</td><td colspan="2">MSG</td><td colspan="2">GOES-16/-17</td><td colspan="2">FY-4A</td><td colspan="2">Geo-KOMPSAT-2A</td><td colspan="2">Himawari-8/-9</td><td colspan="2">MTG-I</td></tr>
<tr><td>Satellite Position</td><td colspan="2">3.4° W</td><td colspan="2">75.2° W (16)
137.2° W (17)</td><td colspan="2">105° E</td><td colspan="2">128.2° E</td><td colspan="2">140.7° E</td><td colspan="2">To be determined</td></tr>
<tr><td>Band Center (μm) & Resolution (km)</td><td>-
-
.635
.810</td><td>-
-
3
3</td><td>.470
-
.640
.865</td><td>1
-
.5
1</td><td>.470
-
.650
.825</td><td>1
-
.5
1</td><td>.470
.509
.640
.865</td><td>1
1
.5
1</td><td>.470
.510
.640
.857</td><td>1
1
.5
1</td><td>.444
.510
.640
.865</td><td>1
1
.5
1</td></tr>
<tr><td>Temporal Resolution (Full Disk)</td><td colspan="2">15 min</td><td colspan="2">15 min</td><td colspan="2">15 min</td><td colspan="2">10 min</td><td colspan="2">10 min</td><td colspan="2">10 min</td></tr>
<tr><td>First Launch Date</td><td colspan="2">August 2002</td><td colspan="2">November 2016</td><td colspan="2">December 2016</td><td colspan="2">December 2018</td><td colspan="2">October 2014</td><td colspan="2">Launch in 2021</td></tr>
</table>

Supplementary Table S2. Phenological Transition Dates (DOYs) Estimated from *In Situ* Time-lapse Digital Images

**Site**	**Phenological Events**	**2016**	**2017**	**Mean**
Takayama (TKY)	Start of Leaf Expansion	125	131	128
	End of Leaf Expansion	150	153	151.5
	Start of Leaf Fall	280	272	276
	End of Leaf Fall	325	319	322
Fujihokuroku (FHK)	Start of Leaf Expansion	105	108	106.5
	End of Leaf Expansion	136	140	138
	Start of Leaf Fall	280	283	281.5
	End of Leaf Fall	329	334	331.5

Supplementary Table S3. Comparison of Number of Days with Cloud-free Observations between AHI and VIIRS. The percent values were obtained by dividing the number of days with cloud-free observations by the number of days with PEN sky images, whereas the frequency values were calculated by dividing the latter by the former.

(a) Takayama (TKY) for Year 2016

Period	Range (DOY)	Total No. of Days	No. of Days with PEN		No. of Days with Cloud-free Obs.		Percentage (%)		Frequency	
			AHI	VIIRS	AHI	VIIRS	AHI	VIIRS	AHI	VIIRS
Pre-green-up	1-124	124	124	124	35	19	28	15	3.5	6.5
Green-up	125-150	26	26	26	7	4	27	15	3.7	6.5
Peak	151-279	129	129	129	14	5	11	3.9	9.2	26
Brown-down	280-325	46	45	45	15	11	33	24	3.0	4.1
Post-brown-down	326-366	41	41	41	8	8	20	20	5.1	5.1

(b) TKY for Year 2017

Period	Range (DOY)	Total No. of Days	No. of Days with PEN		No. of Days with Cloud-free Obs.		Percentage (%)		Frequency	
			AHI	VIIRS	AHI	VIIRS	AHI	VIIRS	AHI	VIIRS
Pre-green-up	1-130	130	130	130	27	15	21	12	4.8	8.7
Green-up	131-153	23	23	23	6	4	26	17	3.8	5.8
Peak	154-271	118	118	118	15	10	13	8.5	7.9	12
Brown-down	272-319	48	48	48	15	9	31	19	3.2	5.3
Post-brown-down	320-365	46	46	46	9	7	20	15	5.1	6.6

(c) Fujihokuroku (FHK) for Year 2016

Period	Range (DOY)	Total No. of Days	No. of Days with PEN		No. of Days with Cloud-free Obs.		Percentage (%)		Frequency	
			AHI	VIIRS	AHI	VIIRS	AHI	VIIRS	AHI	VIIRS
Pre-green-up	1-104	104	100	99	36	18	36	18	2.8	5.5
Green-up	105-136	32	32	32	6	4	19	13	5.3	8.0
Peak	137-279	143	141	141	6	1	4.3	.71	24	141
Brown-down	280-329	50	49	49	13	10	27	20	3.8	4.9
Post-brown-down	330-366	37	35	34	21	14	60	41	1.7	5.5

(Supplementary Table S3 – continued)

(d) FHK for Year 2017

Period	Range (DOY)	Total No. of Days	No. of Days with PEN		No. of Days with Cloud-free Obs.		Percentage (%)		Frequency	
			AHI	VIIRS	AHI	VIIRS	AHI	VIIRS	AHI	VIIRS
Pre-green-up	1-107	107	107	107	44	25	41	23	2.4	4.3
Green-up	108-140	33	33	33	5	3	15	9.1	6.6	11
Peak	141-282	142	142	142	3	2	2.1	1.4	47	71
Brown-down	283-334	52	52	52	22	12	42	23	2.4	4.3
Post-brown-down	335-365	31	30	29	23	16	77	55	1.3	1.8

(e) Terrestrial Environment Research Center of University of Tsukuba (TGF) for Year 2016

Period	Range (DOY)	Total No. of Days	No. of Days with PEN		No. of Days with Cloud-free Obs.		Percentage (%)		Frequency	
			AHI	VIIRS	AHI	VIIRS	AHI	VIIRS	AHI	VIIRS
Pre-green-up	1- 90	90	90	90	47	37	52	41	1.9	2.4
Green-up	91-135	45	45	45	11	5	24	11	4.1	9.0
Peak	136-240	105	102	101	14	3	14	3.0	7.3	34
Brown-down	241-330	90	76	75	23	17	30	23	3.3	4.4
Post-brown-down	331-366	36	36	36	24	16	67	44	1.5	2.3

(f) TGF for Year 2017

Period	Range (DOY)	Total No. of Days	No. of Days with PEN		No. of Days with Cloud-free Obs.		Percentage (%)		Frequency	
			AHI	VIIRS	AHI	VIIRS	AHI	VIIRS	AHI	VIIRS
Pre-green-up	1- 90	90	90	89	53	32	59	36	1.7	2.8
Green-up	91-135	45	42	41	9	6	21	15	4.7	6.8
Peak	136-240	105	104	104	7	2	6.7	1.9	15	52
Brown-down	241-330	90	90	90	25	18	28	20	3.6	5.0
Post-brown-down	331-365	35	34	34	25	21	74	62	1.4	1.6

Supplementary Figure S1. Locations of three study sites, Takayama (TKY), Fujihokuroku (FHK), and Terrestrial Environment Research Center of University of Tsukuba (TGF), superimposed on Himawari AHI NDVI image. This NDVI image was generated by temporally compositing all AHI NDVI images acquired in the month of July 2016 with the maximum-value compositing method. The coastline vector dataset was obtained from the Geospatial Information Authority of Japan (http://www.gsi.go.jp/kankyochiri/gm_japan_e.html). The map was created with ArcGIS® software (ArcMap™ 10.6.1, Esri, Redlands, California, USA, http://www.esri.com).

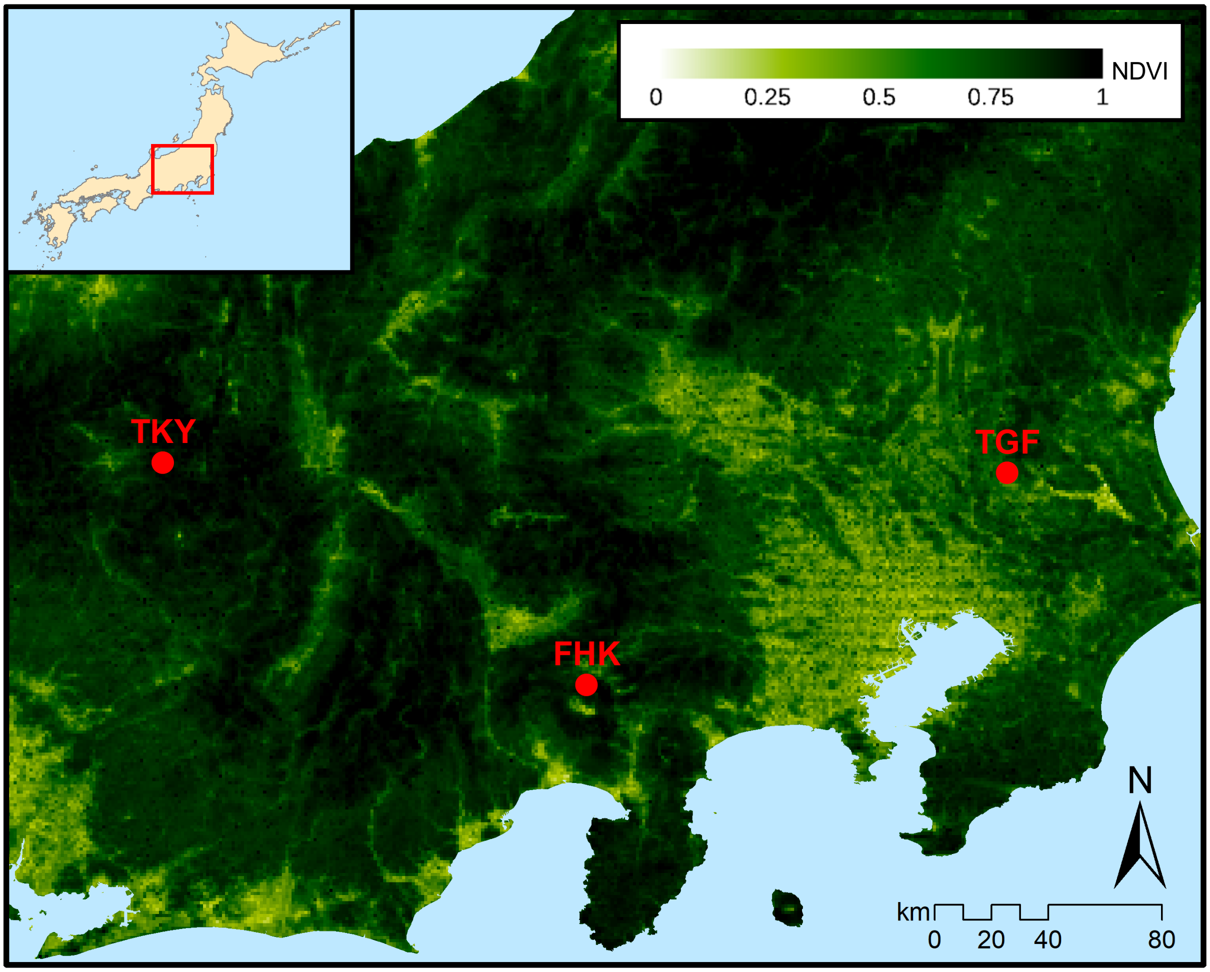

Supplementary Figure S2. AHI NDVI temporal profile (middle) compared to the VIIRS counterpart (bottom) for the Takayama (TKY) site for the year 2017. Sample PEN *in situ* images representing five phenological stages are shown at the top. The numbers on the PEN images are the year (lower-left) and DOY (lower-right) of the corresponding image acquisition. The vertical dashed lines are phenological transition dates identified with PEN time-lapse images (see Supplementary Table S2 online). Low NDVI values indicated by "SG1" were associated with persistent cloud cover during the baiu season followed by the passage of Typhoon-3 and those indicated by "SG2" were due to thick cloud cover followed by the passage of Typhoon-21.

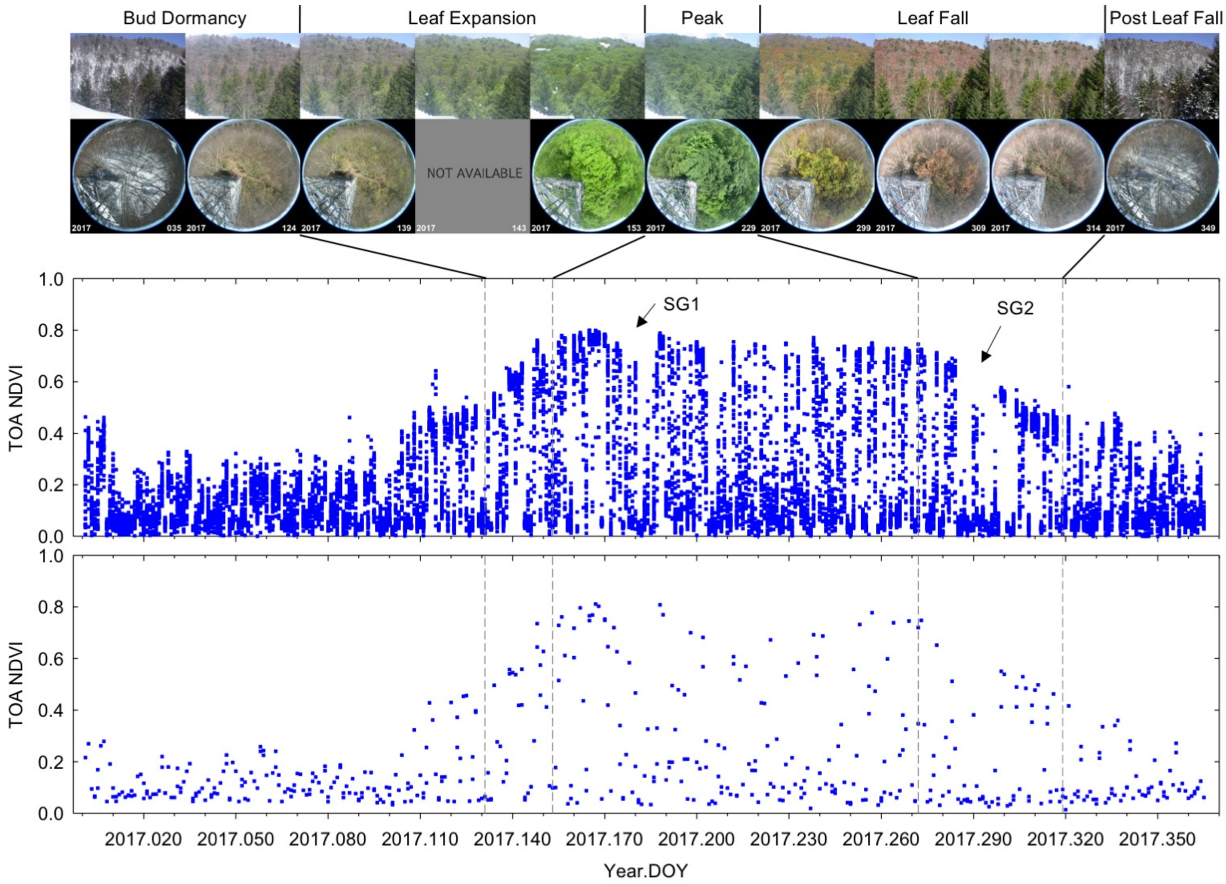

Supplementary Figure S3. AHI NDVI seasonal changes (middle) during the first five months of the year 2017 for the Fujihokuroku (FHK) site. Plotted at the bottom is the VIIRS counterpart for comparison. Representative PEN *in situ* images for every distinctive snow cover condition are shown at the top. The numbers on the PEN images are the year (lower-left) and DOY (lower-right) of the corresponding image acquisition. The AHI NDVI was continuously higher during a period indicated by "SC1" than during nearby pre- and post-SC1 periods, which was not clearly visible in the VIIRS NDVI. PEN images showed receded snow cover for the SC1 period.

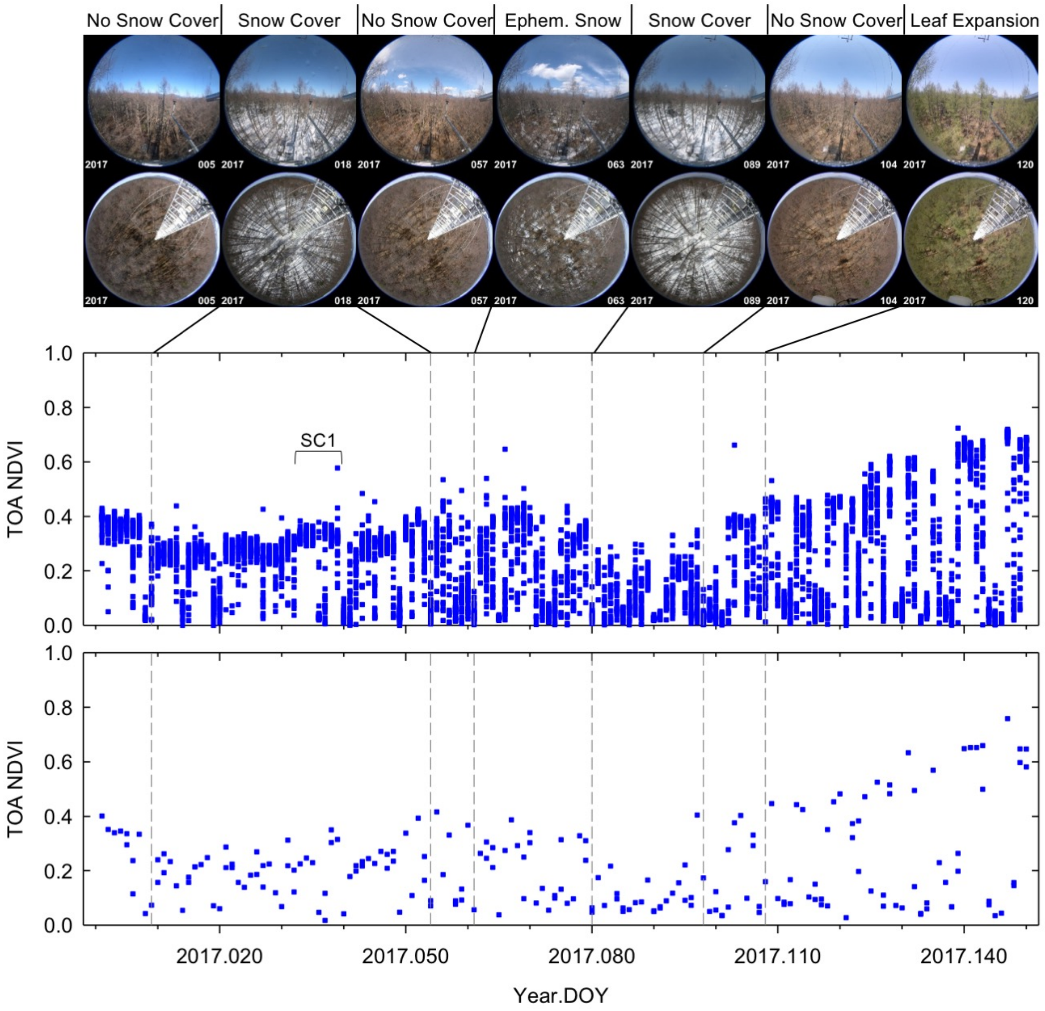

Supplementary Figure S4. VIIRS and AHI NDVI data over spring green-up and fall brown-down periods for the Takayama (TKY) site for the year 2017: (a) VIIRS and (b) AHI for the green-up season, and (c) VIIRS and (d) AHI for the brown-down period.

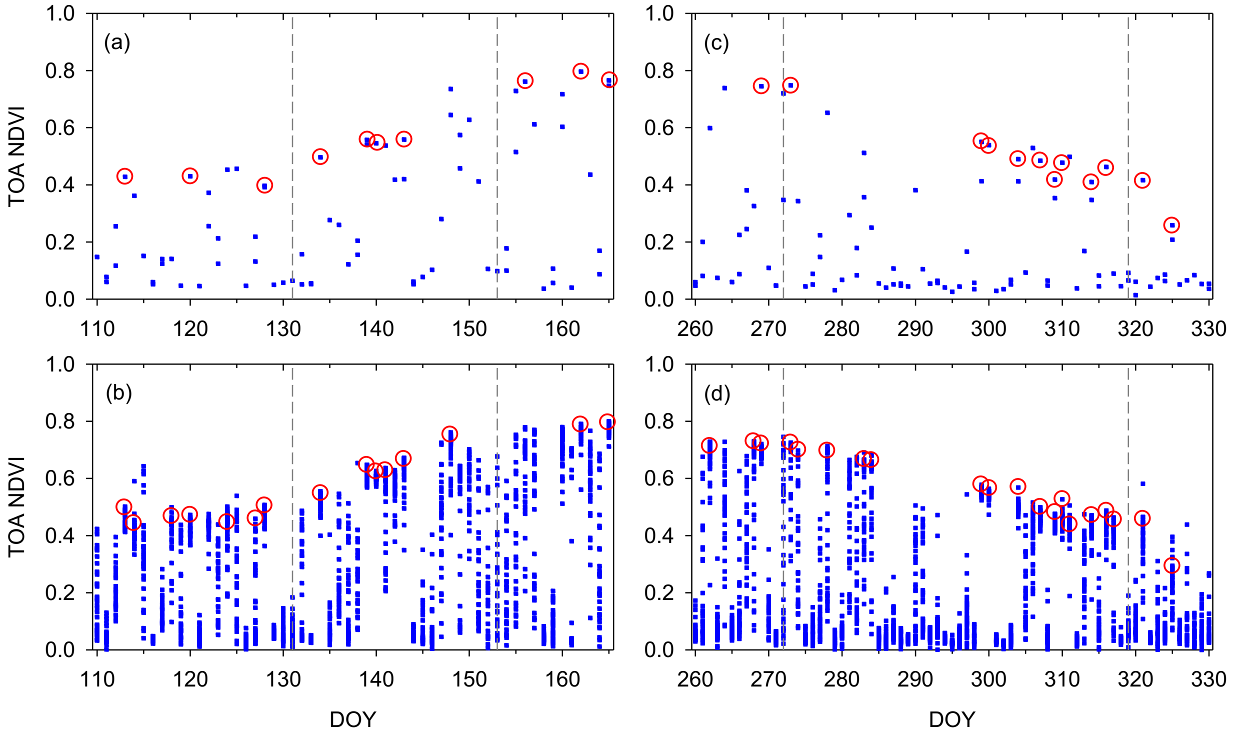

Supplementary Figure S5. Same as Supplementary Fig. S4, but for the Fujihokuroku (FHK) site for the year 2016.

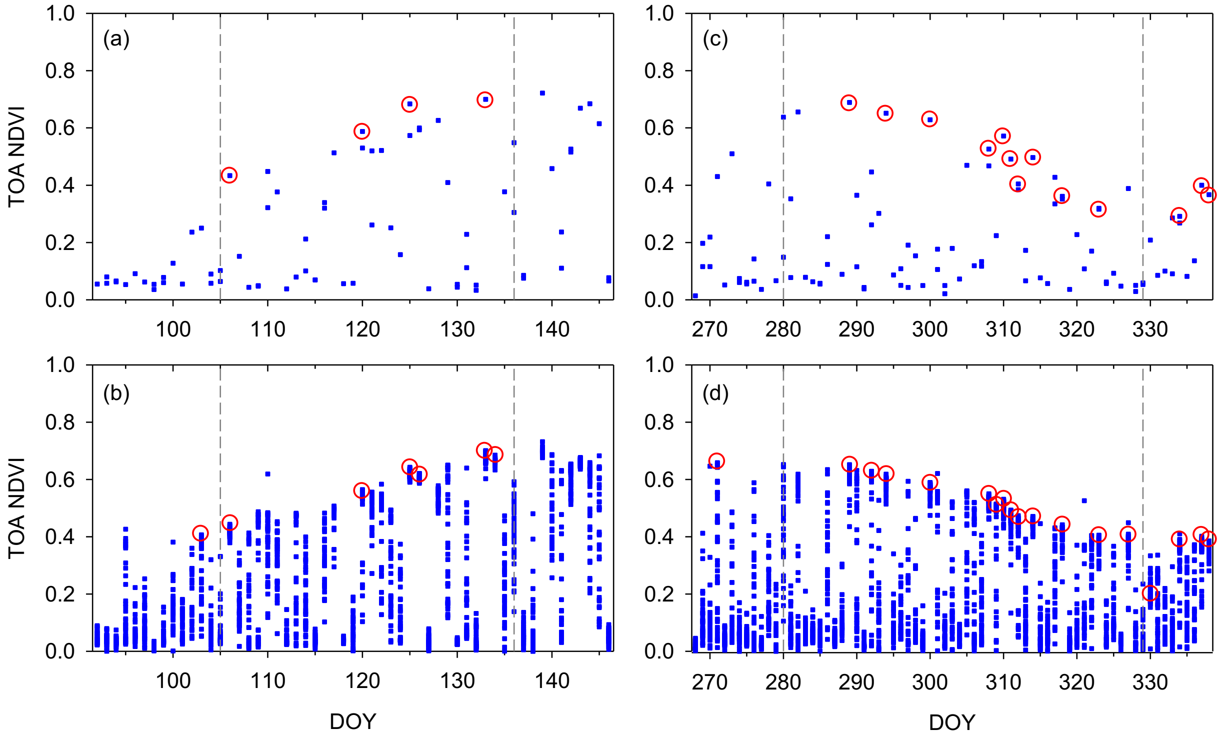

Supplementary Figure S6. Same as Supplementary Fig. S4, but for the FHK site for the year 2017.

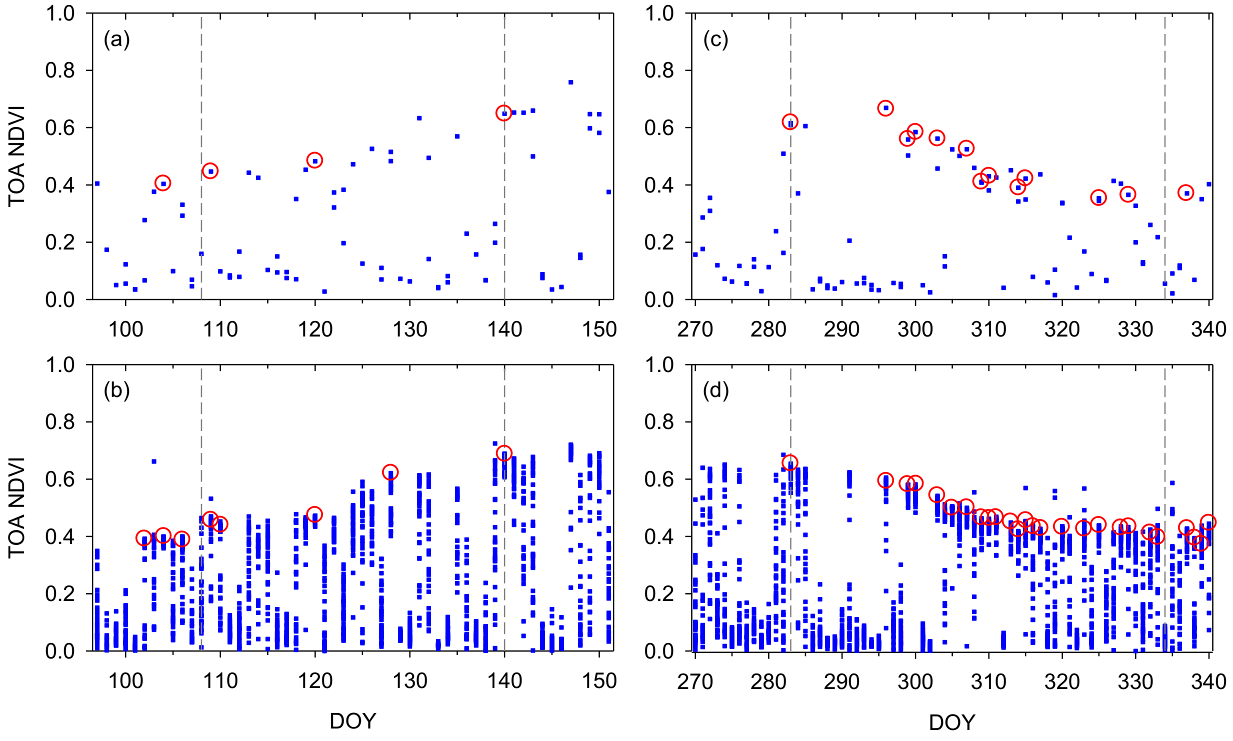

Supplementary Figure S7. Same as Supplementary Fig. S4, but for the Terrestrial Environment Research Center of University of Tsukuba (TGF) site for the year 2016. The green-up and brown-down periods for the TGF site were determined to enclose the periods of the increasing and decreasing NDVI, respectively, as observed in the AHI and VIIRS temporal profiles extracted over the site (see Fig. 1e, f).

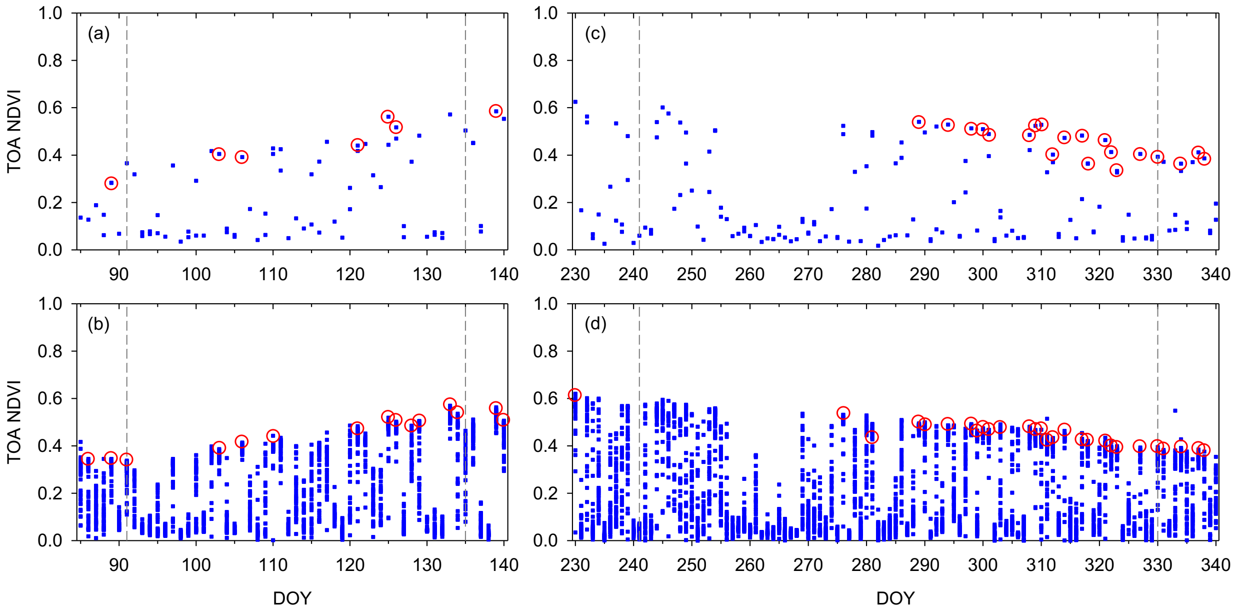

Supplementary Figure S8. Same as Supplementary Fig. S4, but for the TGF site for the year 2017.

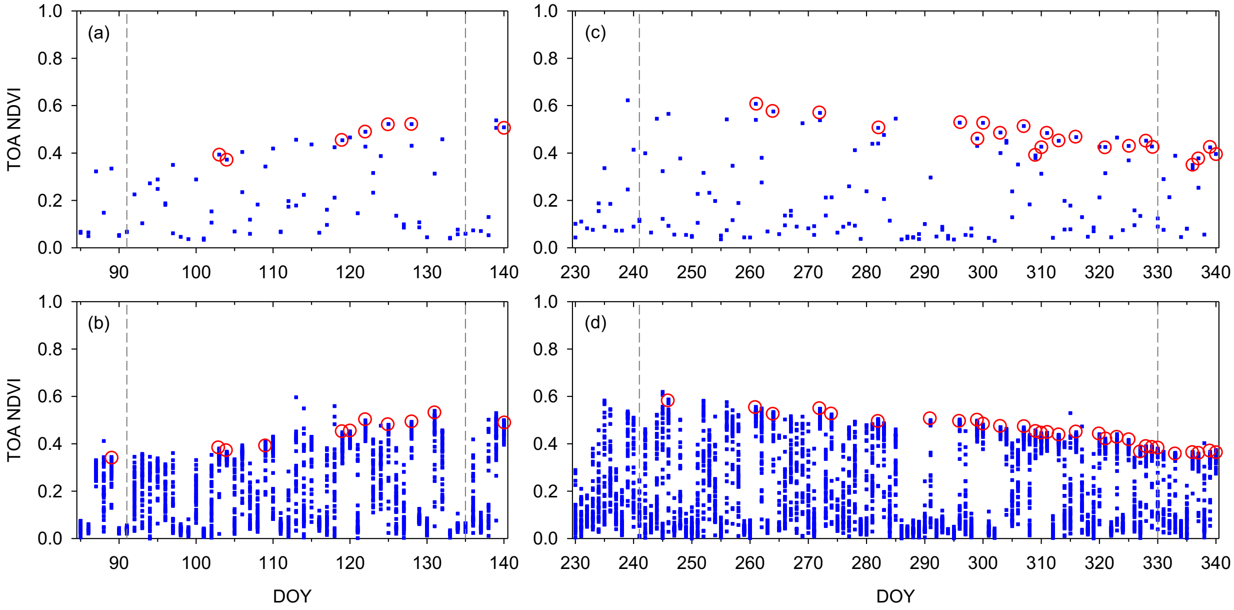

Supplementary Figure S9. AHI NDVI diurnal time series plots of select pairs of days for the Takayama (TKY) site. Each plot contains two time series: one without any confirmed "cloud-free" observations (red circles) and the other with confirmed cloud-free observations (blue diamonds) by PEN sky images. The blue open diamonds represent confirmed "cloud-free" observations and, thus, cloud-free NDVI values. The brackets indicate the time periods where visual inspection of AHI false color composite images indicated cloud-free AHI observations over the TKY site. For each plot, one sample AHI false color image during the time period (the time of the image indicated by the arrow on the plot) is shown at the right of the plot.

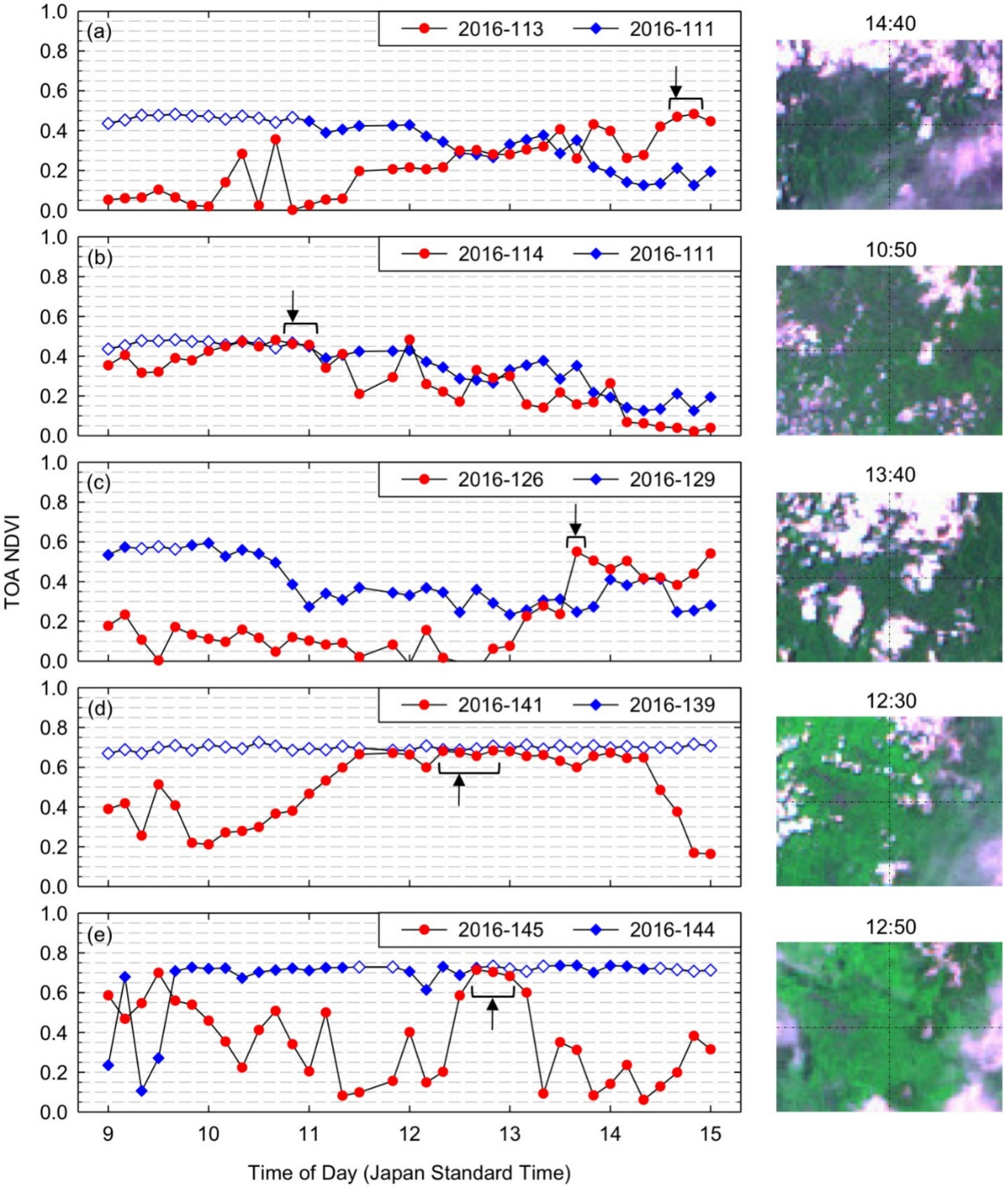

(Supplementary Figure S9 – continued)

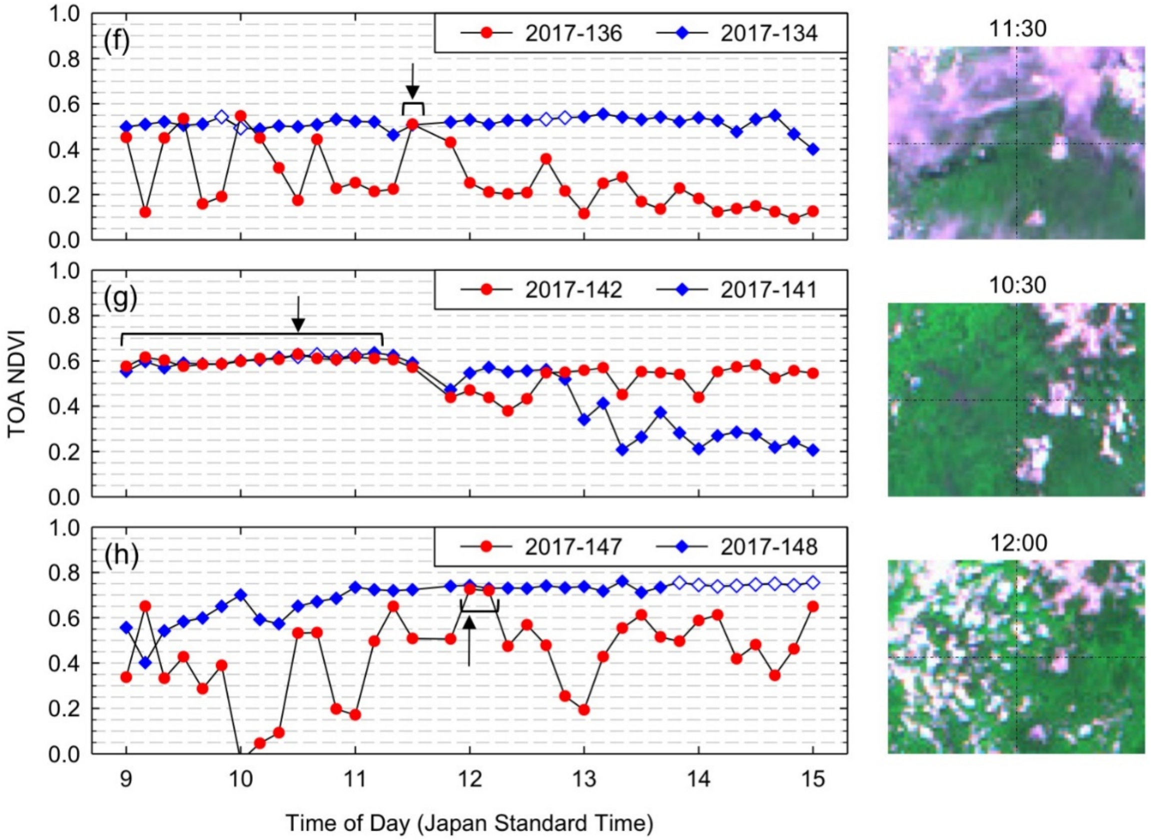

Supplement: Supplementary file 1 — Supplementary Information [file 41598_2019_52076_MOESM1_ESM.pdf]
